# Static Baropodometric Assessment for Musculoskeletal Rehabilitation: Plantar Pressure and Postural Load Distribution in Young Adults

**DOI:** 10.3390/life15091354

**Published:** 2025-08-26

**Authors:** Tudor Vladimir Gurau, Madalina Gabriela Coman, Daniel Andrei Iordan, Ilie Onu, Cosmin Raducu Raileanu, Andreea Maria Adam, Gabriela Gurau, Doina Carina Voinescu, Adela Badau, Carmina Liana Musat

**Affiliations:** 1 Faculty of Medicine and Pharmacy, “Dunarea de Jos” University of Galati, 800008 Galati, Romania; tudor.gurau@ugal.ro (T.V.G.); cosmin.raileanu@ugal.ro (C.R.R.); gabriela.gurau@ugal.ro (G.G.); carinavoinescu@gmail.com (D.C.V.); carmina.musat@ugal.ro (C.L.M.); 2 Faculty of Physical Education and Sport, “Dunarea de Jos” University of Galati, 800008 Galati, Romania; madalina.postelnicu@ugal.ro (M.G.C.); andreea.adam@ugal.ro (A.M.A.); 3 Center of Physical Therapy and Rehabilitation, “Dunarea de Jos” University of Galati, 800008 Galati, Romania; 4 Faculty of Medical Bioengineering, University of Medicine and Pharmacy “Grigore T. Popa” Iasi, 700454 Iasi, Romania; 5 Faculty of Physical Education and Mountain Sports, Transilvania University of Brasov, 500068 Brasov, Romania; adela.badau@unitbv.ro

**Keywords:** physical rehabilitation, musculoskeletal disorders, plantar pressure, baropodometry, postural balance, mobility enhancement, foot biomechanics, physical therapy, manual therapy

## Abstract

Plantar pressure and foot load distribution are essential parameters in evaluating postural alignment, neuromuscular balance, and the risk of musculoskeletal disorders. This cross-sectional study analyzed static plantar pressure in 113 healthy young adults (18–35 years) using the Spine 3D Sensor Medica platform. Contact area, load, average pressure, and maximum peak pressure were measured bilaterally in forefoot and hindfoot regions. Strong correlations were found between body weight, BMI, and plantar load, particularly in the hindfoot, while peak pressures were influenced more by individual biomechanical factors than anthropometry. Women demonstrated greater inter-limb asymmetries compared to men. These findings provide reference values for static plantar pressure and highlight their importance for clinical musculoskeletal rehabilitation. By identifying early postural imbalances and abnormal load distributions, baropodometric assessments can guide targeted interventions, improve pain management, and optimize functional recovery. The study supports incorporating advanced plantar pressure analysis in personalized rehabilitation programs for preventing and managing musculoskeletal dysfunctions.

## 1. Introduction

Postural balance and the efficient distribution of plantar pressure are fundamental components of neuromuscular function, playing a critical role in maintaining stability, preventing biomechanical dysfunctions, and enhancing motor performance. Plantar pressure, defined as the force exerted by the foot on the ground per unit area, serves as a key biomechanical parameter in both static and dynamic evaluations of gait, posture, and balance [1].

Computerized baropodometry has emerged as a reliable, non-invasive method for assessing plantar load distribution under various conditions. This technology enables the measurement of crucial indicators such as contact area, body weight distribution, average plantar pressure, and peak pressure in distinct foot regions (forefoot and hindfoot), providing clinically relevant insights for diagnosis, monitoring, and personalized therapeutic interventions in physical therapy and rehabilitation [2,3].

The distribution of plantar pressure is shaped by various factors, thereby maintaining a balance of factors, including body mass, body mass index (BMI), height, foot morphology, postural alignment, gait characteristics, and neuromuscular control [4,5,6]. Postural stability refers to the ability of the Postural Control System (PCS)—a complex network that integrates central nervous system structures with peripheral sensory receptors—to maintain the vertical projection of the center of mass within the base of support [7,8]. These components interact synergistically to modulate myofascial chains against gravitational forces and maintain balance during various motor tasks. Proper physiological distribution of plantar pressure plays a key role in this process and is essential for identifying and managing potential musculoskeletal dysfunctions. Recent studies have shown that postural stability and plantar pressure parameters vary depending on visual conditions (e.g., eyes open vs. closed) and viewing distances, highlighting the importance of sensory input in postural regulation [9,10].

Emerging evidence also suggests that foot dominance, often linked to handedness, may affect plantar loading symmetry and contribute to inter-limb discrepancies [11,12]. Moreover, the symmetry between the lower limbs—particularly in terms of plantar loading—is not purely anatomical but is shaped by genetic predispositions, neuromuscular patterns, limb preference (typically right-side dominance), and external factors such as fatigue. Dominant limbs tend to exhibit greater muscular development and altered kinematic profiles, which can lead to measurable differences in plantar pressure and load distribution [13,14]. These asymmetries can be exacerbated by prolonged physical effort, potentially reducing movement efficiency and increasing injury risk [15,16,17]. Therefore, the evaluation of plantar symmetry is critical not only in biomechanical profiling but also in the design of preventive and rehabilitative strategies [18,19,20].

Despite growing interest in baropodometric evaluation, few studies have utilized advanced 3D-integrated platforms such as the Spine 3D Sensor Medica system, which combines static baropodometric measurements with three-dimensional postural analysis [21,22,23,24,25]. There remains a need to describe and validate the technical capabilities (e.g., sensitivity, accuracy) of such systems to promote standardization in biomechanical evaluations [26,27,28,29].

In this context, the present study aims to investigate static baropodometric parameters in a population of healthy young adults aged 18 to 35 years. The analysis focuses on contact area, absolute and relative load, average pressure, and maximum peak pressure in both the forefoot and hindfoot regions. The specific objectives are to:

- examine the relationships between plantar pressure parameters and anthropometric variables (age, weight, height, BMI).
- identify potential asymmetries between the right and left feet.
- evaluate the influence of BMI on plantar pressure distribution.

The findings of this study are intended to establish normative reference values for healthy young adults and to support personalized approaches in rehabilitation, posture correction, and injury prevention.

## 2. Materials and Methods

### 2.1. Study Design and Participants

This is a cross-sectional observational study conducted between November 2024 and March 2025 at the University Center for Physiotherapy and Medical Rehabilitation, “Dunarea de Jos” University of Galati, Romania. The study was approved by the institutional ethics committee (approval no. 20/9 December 2024), and written informed consent was obtained from all participants in accordance with the Declaration of Helsinki.

**Figure 1:**
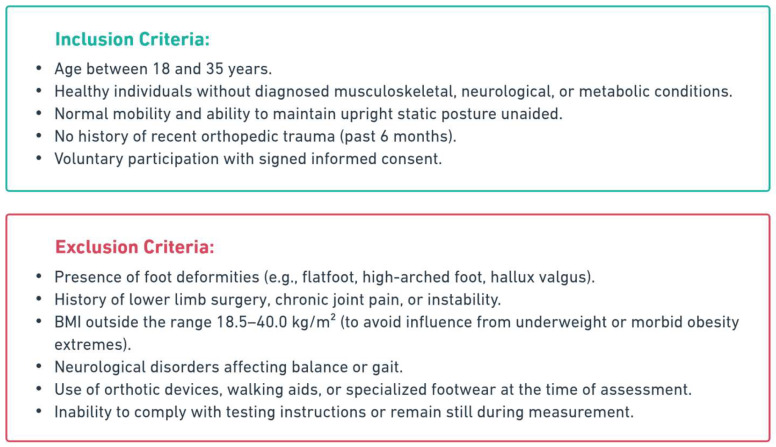
Flowchart illustrating participant recruitment, inclusion/exclusion process.

A total of 113 young adults (53 women and 60 men) aged between 18 and 35 years were included. Participants were recruited from among students enrolled in health and sports science faculties. A flowchart of participant selection is illustrated in Figure 1.

### 2.2. Anthropometric Assessment

All participants underwent anthropometric assessment prior to baropodometric testing. Measurements were taken using a medical scale with an integrated stadiometer, calibrated according to institutional standards. For each subject, body weight (expressed in kilograms, kg) and height (expressed in meters, m) were recorded. Based on this data, the body mass index (BMI) was calculated using the classic formula:

(1) $$\mathrm{BMI}\;=\;\frac{Weight\;(kg)}{{Height}^{2}\;(m^{2})},$$

The BMI value was used to classify participants into the following categories, according to the World Health Organization (WHO) classification [30]: underweight (<18.5 kg/m^2^), normal weight (18.5–24.9 kg/m^2^), overweight (25.0–29.9 kg/m^2^), obesity class I (30.0–34.9 kg/m^2^), obesity class II (35.0–39.9 kg/m^2^) and obesity class III (≥40 kg/m^2^). Participants who did not fall within the acceptable range (BMI below 18.5 or above 40) were excluded from the analysis to avoid any extreme effects on the distribution of plantar pressure.

Anthropometric results were presented separately for women and men, including the mean, standard deviation (SD), and minimum and maximum ranges for each variable (age, weight, height, and BMI), as shown in Table 1. These data were then correlated with baropodometric parameters to identify possible significant relationships between body characteristics and plantar pressure distribution.

### 2.3. Baropodometric Assessment

Plantar pressure assessment was performed using the integrated Spine 3D Sensor Medica system (Sensor Medica, Albuccione, Italy), which combines a baropodometric platform with three-dimensional analysis of posture and static balance [31]. The system is validated for clinical and research applications, with high repeatability and reliability for static plantar pressure mapping, as previously reported in similar biomechanical studies [32]. This technology is widely used in clinical biomechanics for both static and dynamic assessments of the foot and axial posture [33]. The platform has a resolution of 4 sensors/cm^2^, a sampling frequency of 100 Hz, and a pressure measurement capacity of up to 1200 kPa. The system was calibrated before each test session according to the standard protocol provided by the manufacturer.

The testing was conducted in a controlled laboratory environment, under constant lighting and stable room temperature. Participants stood barefoot in a relaxed posture with both feet symmetrically aligned on the platform, arms at their sides, and gaze fixed forward. Each assessment included three consecutive static trials, each lasting approximately 10 s, from which average values were calculated for accuracy.

For each foot (right and left), data were segmented into two anatomical regions—the forefoot (metatarsal area) and hindfoot (calcaneal area). The main parameters analyzed included: contact area (cm^2^), absolute and relative load (kgf and % body weight), average pressure (kPa), and maximum peak pressure (kPa). These metrics were later compared across sexes, BMI groups, and anatomical sides.

**Figure 2:**
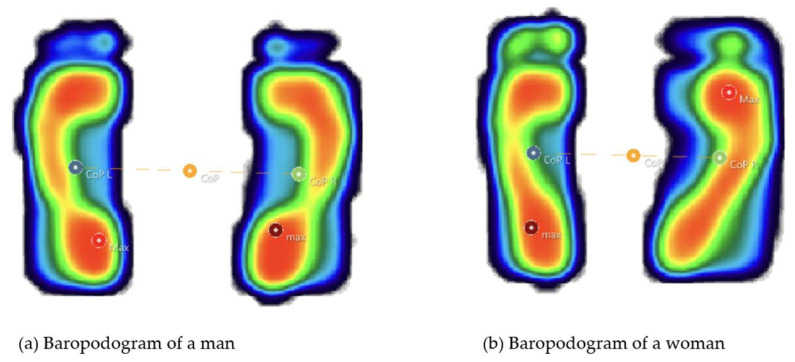
Comparative static baropodograms in male (**a**) and female (**b**) subjects.

**Figure 3:**
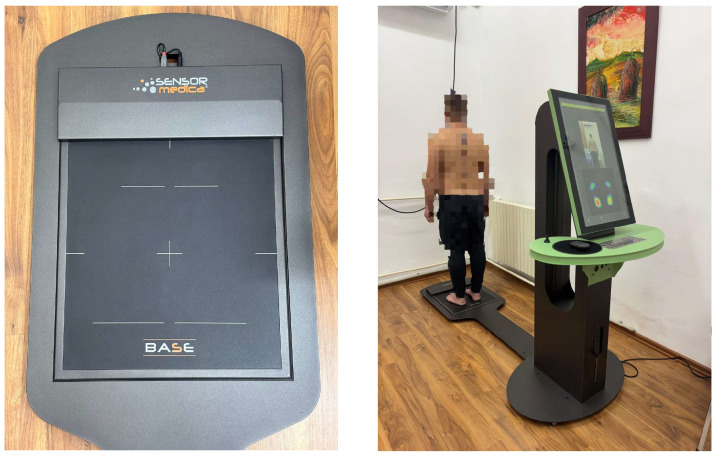
Experimental setup with the Spine 3D Sensor Medica system.

Figure 2 presents a comparative example of static baropodograms between a male and a female participant, while Figure 3 displays the actual laboratory setup and equipment configuration used for the study.

### 2.4. Testing Protocol

Each participant was assessed individually in a room dedicated to baropodometric testing, in a constant temperature environment, without noise or visual distractions. The subjects were instructed to stand barefoot, in a relaxed and symmetrical position, with their feet parallel and shoulder-width apart, their arms hanging loosely at their sides, and their gaze directed horizontally toward a fixed point on the wall. To minimize postural compensation, the head was positioned neutrally, and the knees were kept extended but not locked. Feet were aligned shoulder-width apart with equal support on both limbs. Correct positioning on the pressure platform was supervised by a trained evaluator to avoid alignment errors that could alter the biomechanical data.

The assessments were carried out in a static position, without any voluntary movements on the part of the participant. For each person, three successive recordings were made, each lasting 10 s, with a 30-s rest period between them. Subsequently, the average values for each parameter analyzed were calculated, thus ensuring data consistency.

Within each recording, the system measured the following baropodometric parameters for each foot (right and left), respectively, for the two anatomical regions of the foot: forefoot and hindfoot:

- contact area (cm^2^).
- absolute load (kgf) and relative load (% of body weight).
- average pressure (kPa).
- maximum peak pressure (kPa).

These indicators reflect the load distribution and functional adaptations of the foot during standing. All values were automatically stored by the Spine 3D software (https://spine-3d.com/) and were subsequently exported for statistical analysis, as detailed in Table 2.

### 2.5. Data Processing and Symmetry Analysis

The data collected through the baropodometric platform was processed using the Motion Tools and Spine 3D software suite, specific to the equipment used. This allowed for the automatic segmentation of the foot into the two regions of interest (forefoot and hindfoot), as well as the numerical calculation of the values for each biomechanical parameter. For each participant, the data were analyzed both individually and comparatively between the right and left foot.

To assess the functional balance between the lower limbs, the asymmetry index (AI) was calculated using the following formula:

(2) $$\mathrm{AI}\;=\;\left(\frac{\left|Right-Left\right|}{0.5\times (Right+Left)}\right)\times 100,$$

The AI value expresses the degree of imbalance between the two legs as a percentage, and its interpretation was made according to the following reference thresholds: values below 10% were considered acceptable functional asymmetry, values between 10% and 20% indicated moderate asymmetry, and values above 20% were classified as significant asymmetry, possibly associated with postural imbalances, unilateral dominance, or subclinical pathologies.

This index was calculated for each of the main parameters (contact area, load, average pressure, and maximum pressure), and the results were compared between genders and BMI categories to identify possible patterns of asymmetry correlated with anthropometric characteristics. The parameters and corresponding thresholds for asymmetry interpretation are summarized in Table 3.

### 2.6. Statistical Analysis

Statistical analysis was performed using IBM SPSS Statistics version 26.0 (IBM Corp., Armonk, NY, USA). Before performing comparative or correlation analyses, all quantitative variables were tested for normal distribution using the Shapiro–Wilk test. This step was essential for choosing the appropriate statistical tests, depending on the nature of the parameters and their distribution.

For continuous variables with normal distribution, data were presented as mean ± standard deviation (SD), while for those with non-parametric distribution, median and interquartile range (IQR) were used. Categorical variables (e.g., BMI categories) were expressed as absolute frequencies and percentages (%).

The comparison of differences between sexes was performed using the *t*-test for independent samples (for normally distributed data) or the Mann–Whitney U test (for non-parametric distributions). Differences between the right and left legs (intra-individual comparisons) were analyzed using the *t*-test for paired samples or the Wilcoxon signed-rank test, depending on normality.

To assess differences between the five BMI categories, one-way ANOVA (for normal and homogeneous data) or Kruskal–Wallis test (for non-normal or unequal variance data) was used, followed by Bonferroni or Dunn post hoc tests, where appropriate.

The relationships between anthropometric variables (age, weight, height, BMI) and baropodometric parameters (contact area, load, pressure) were investigated using Pearson correlations (for parametric data) or Spearman correlations (for non-parametric data). The level of significance was set at *p* < 0.05. For statistically significant analyses, effect sizes were also calculated: Cohen’s d (for differences between two groups) or η^2^ (eta squared) (for ANOVA), to estimate the magnitude of the effect.

This rigorous statistical approach allowed not only the identification of significant differences between groups or conditions, but also the assessment of the strength of relationships between variables, thus contributing to a complex and clinically and biomechanically relevant interpretation.

## 3. Results

### 3.1. Participant Characteristics

A total of 113 healthy participants were enrolled in this study, of whom 60 were men (53.1%) and 53 were women (46.9%). The overall mean age was 21.07 ± 3.19 years (range: 18–35 years), with no statistically significant difference between sexes (men: 21.00 ± 2.33; women: 21.15 ± 3.97). The majority of both male and female participants were aged between 18 and 20 years—61.7% and 66.0%, respectively—reflecting the age distribution of university students. Women had a slightly higher proportion of individuals above 24 years (9.6%) compared to men (3.3%).

Anthropometric evaluation revealed that male participants had significantly higher weight and height than females. On average, men weighed 76.90 ± 12.06 kg and measured 1.79 ± 0.07 m, whereas women weighed 67.83 ± 17.52 kg and measured 1.65 ± 0.06 m. These differences align with the expected biological variation. Body mass index (BMI) values were comparable between sexes (24.74 ± 6.05 kg/m^2^ in women vs. 23.99 ± 3.52 kg/m^2^ in men), though the greater variability among women reflects a wider range of body types.

A summary of these parameters is provided in Table 4, which serves as the baseline for further analysis of plantar pressure distribution and load symmetry.

### 3.2. BMI Categories and Distribution

BMI classification was conducted according to the World Health Organization guidelines. Most participants were classified as having a normal weight, representing 63.3% of men and 47.2% of women. Underweight status was considerably more frequent among women (11.3%) compared to men (1.7%), while overweight status was slightly more prevalent in men (30%) than in women (26.4%).

Obesity was less common but not negligible. Among men, 5.0% were categorized as obesity class I (BMI 30.0–34.9 kg/m^2^). Among women, 7.6% fell into this same category, and an additional 3.75% were classified as obesity class II or III (BMI ≥ 35 kg/m^2^)—a range not observed in the male subgroup.

**Figure 4:**
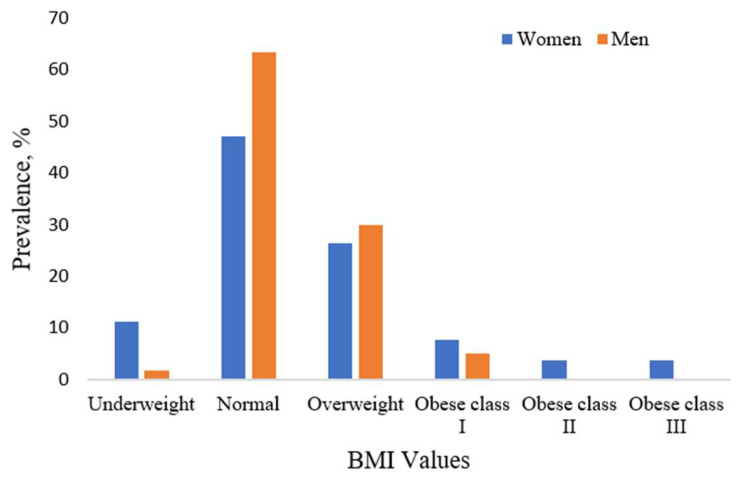
Distribution of participants by sex and BMI category.

These distributions emphasize the anthropometric heterogeneity of the sample and are relevant in interpreting baropodometric parameters. The stratification by sex and BMI category is visually represented in Figure 4.

### 3.3. Surface Distribution

Baropodometric analysis under static conditions revealed consistent anatomical patterns in plantar contact surface and load distribution. These parameters are central to biomechanical and clinical assessments, offering insights into posture, foot mechanics, and potential dysfunctions.

Across the total sample (*n* = 113), the hindfoot exhibited consistently larger contact areas and higher loads than the forefoot. The right hindfoot had the highest average contact area (88.19 cm^2^), while the left forefoot showed the lowest (70.00 cm^2^). This distribution aligns with normal biomechanical expectations, as the heel typically serves as the primary load-bearing region during standing.

In terms of load distribution, the hindfoot absorbed approximately 60% of total body weight, as reflected in mean absolute values: 21.58 kgf (29.52%) for the right hindfoot and 19.94 kgf (27.37%) for the left hindfoot. The forefoot, in contrast, bore 16.55 kgf (22.81%) on the right side and 14.64 kgf (20.27%) on the left. These values confirm a posterior bias in weight distribution during static stance.

Average pressure values ranged between 20.66 and 24.53 kPa, with the hindfoot again displaying the highest figures. Interestingly, although pressure differences were less marked than those of load or contact area, the left hindfoot recorded the highest mean value (24.53 kPa), suggesting subtle postural asymmetries.

Maximum peak pressures, which indicate localized areas of high stress, were also greater in the hindfoot, particularly in the right foot (48.02 kPa). This may reflect structural dominance or habitual stance behavior. The lowest peak was recorded in the left forefoot (43.21 kPa), consistent with reduced contact and load in this region.

**Figure 5:**
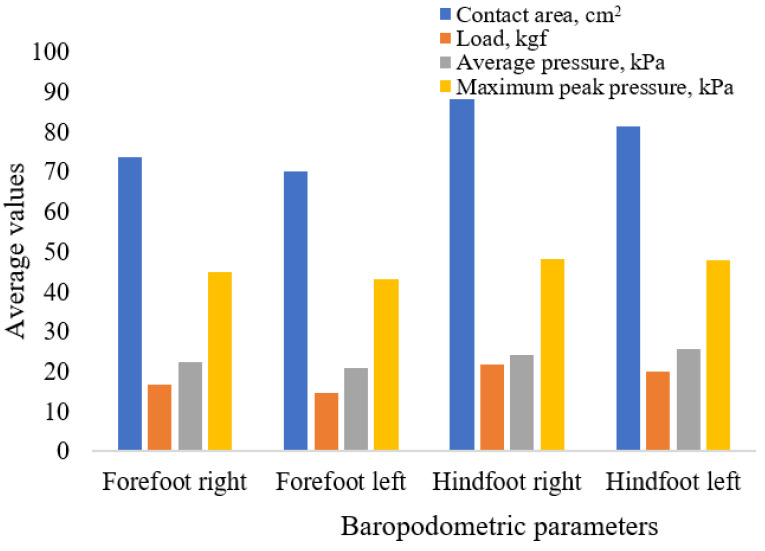
Distribution of baropodometric parameters by foot region (forefoot vs. hindfoot, right vs. left).

A detailed overview of all parameters, including standard deviations and variation ranges, is presented in Table 5. Additionally, Figure 5 provides a graphical representation to facilitate comparisons between anatomical regions.

### 3.4. Contact Area Distribution

Contact area reflects the surface of the foot in contact with the ground and is an essential indicator of postural balance, anatomical morphology, and biomechanical adaptation. In our sample, contact areas were consistently larger in the hindfoot than in the forefoot, a pattern observed in both sexes.

Right–left comparisons revealed more pronounced asymmetries in the hindfoot than in the forefoot. Specifically, the average difference between right and left hindfoot contact areas was 6.15 cm^2^ in men and 7.81 cm^2^ in women, suggesting a mild tendency toward asymmetric rearfoot loading.

When stratified by sex, men exhibited significantly higher contact areas in the hindfoot for both limbs (*p* = 0.006 for RHF and *p* = 0.001 for LHF), while forefoot contact areas did not differ significantly between sexes. The mean hindfoot contact areas were 93.35 cm^2^ (RHF) and 87.20 cm^2^ (LHF) in men, compared to 82.34 cm^2^ and 74.53 cm^2^ in women.

Interestingly, women showed greater asymmetry in the forefoot, with a mean difference of 5.26 cm^2^ between right and left forefoot, compared to 1.91 cm^2^ in men. This pattern may reflect compensatory postural strategies, unilateral dominance, or subclinical structural differences (e.g., leg length discrepancy, ligamentous laxity, or prior minor trauma).

Individual-level analysis (Table 6) revealed greater imbalances among participants with extreme BMI values, with overweight and underweight individuals showing both excessive and insufficient plantar surface distribution, respectively.

### 3.5. Load Distribution

Under normal static conditions, approximately 60% of the body’s weight should be distributed over the hindfoot and 40% over the forefoot. Our findings align with this expected pattern but also revealed notable differences between sexes and individual asymmetries.

In both sexes, the hindfoot consistently supported higher absolute and relative load compared to the forefoot. The highest mean value was observed in the right hindfoot of men (22.78 kgf), while the lowest was recorded in the left forefoot of women (13.53 kgf).

Men showed significantly higher absolute loads across all foot regions compared to women (*p* < 0.01), likely reflecting their greater body weight and muscle mass. However, women demonstrated greater asymmetry, particularly in the hindfoot, where 12 individuals exhibited differences > 4 kgf between limbs. These imbalances were not always associated with BMI, suggesting the role of other factors such as posture, stability, and foot dominance.

Among overweight and obese individuals, both male and female, inter-limb load imbalances were more frequent and more pronounced—reaching differences of up to 14 kgf in some cases. This supports the idea that increased body mass imposes uneven mechanical demands on the lower limbs, potentially predisposing to musculoskeletal issues.

While percentage load values followed the same trend as absolute values, they offered a standardized perspective independent of body weight and also confirmed the predominance of hindfoot support during upright stance.

These results (Table 7) underline the importance of evaluating both absolute and relative load in clinical and ergonomic contexts, particularly for populations at risk of postural deviations or overuse injuries.

### 3.6. Average Pressure Distribution

Average plantar pressure reflects the mean pressure exerted by each foot region during stance. In our study, men had significantly higher mean pressures at the forefoot level (*p* = 0.030 for RFF and *p* = 0.046 for LFF), while no significant sex differences were observed in the hindfoot. Pressures were generally higher in the hindfoot compared to the forefoot across both sexes. These differences may be linked to foot posture, body composition, or gait habits.

In women, forefoot pressures varied more widely and correlated moderately with BMI. Table 8 presents average pressures by sex and region.

Average plantar pressure quantifies the mean force per unit area exerted by each foot region during static stance, serving as an indicator of load distribution and potential biomechanical inefficiencies. In this study, mean pressure values were generally higher in the hindfoot than in the forefoot for both sexes, consistent with the physiological trend of posterior load dominance in upright posture.

Statistically significant differences between sexes were observed at the forefoot level: men exhibited higher average pressures than women in both the right forefoot (*p* = 0.030) and left forefoot (*p* = 0.046). No significant sex-related differences were found in the hindfoot.

Interestingly, in women, forefoot pressure values exhibited greater variability, with individual differences ranging from 0 to 13 kPa. These values showed moderate correlations with BMI, suggesting that body mass and fat distribution may influence localized foot loading patterns.

At the hindfoot level, pressure values ranged up to 24.53 kPa on the left side, indicating a tendency toward higher posterior loading, particularly in static stance. These pressures may be influenced not only by anatomical structure but also by habitual posture or subtle limb length discrepancies.

### 3.7. Maximum Peak Pressure Distribution

Maximum peak pressure indicates localized pressure concentration, which is relevant for detecting biomechanical dysfunctions and areas prone to stress-related injuries. In the present study, peak pressures were highest in the hindfoot, with greater interindividual variability observed in women.

The only statistically significant difference between sexes was found in the right forefoot, where men showed higher peak pressures (*p* = 0.017). For the remaining regions, although mean values were slightly higher in men, the differences did not reach statistical significance.

Intra-individual analysis revealed several participants with notable right–left differences exceeding 5 kPa, particularly in the hindfoot region. Surprisingly, these variations were not consistently associated with BMI, indicating that intrinsic biomechanical factors, such as gait pattern or structural asymmetries, may have a stronger influence.

The person with the highest recorded peak pressure (82 kPa) also showed the largest asymmetry (34 kPa) in the hindfoot, despite having a low BMI (18.0 kg/m^2^). This suggests that excessive localized force can occur independently of body mass, likely due to postural imbalance or heel-strike dominance.

**Figure 6:**
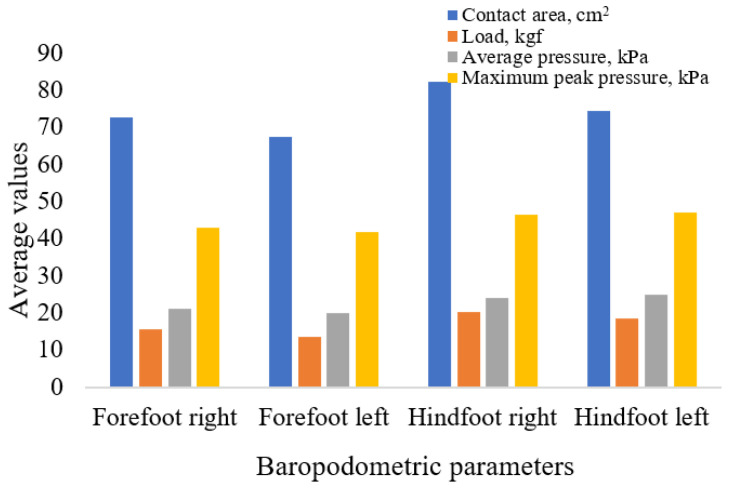
Distribution of baropodometric parameters in the female group.

**Figure 7:**
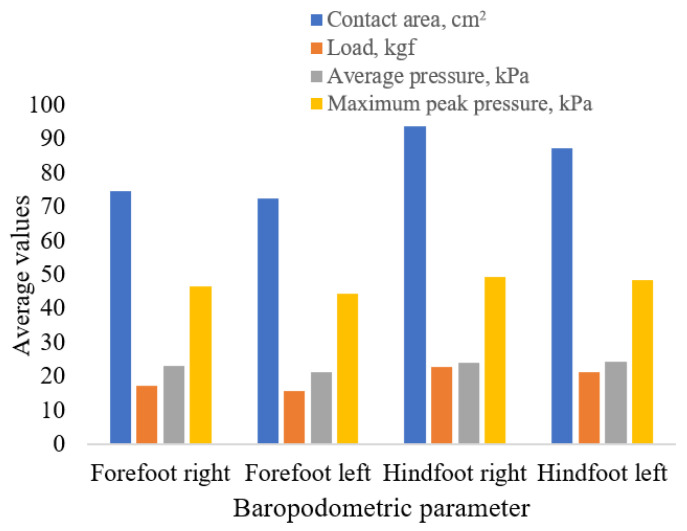
Distribution of baropodometric parameters in the male group.

Figure 6 and Figure 7 illustrate the regional pressure variations for female and male participants, respectively. Table 9 presents the maximum peak pressure values, stratified by sex and foot region.

### 3.8. Distribution Patterns: Skewness and Kurtosis

**Figure 8:**
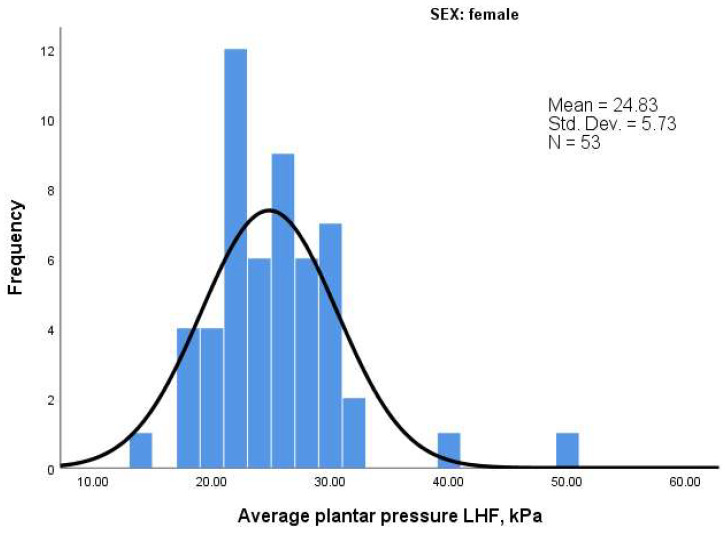
Histogram of Average Pressure in Left Hindfoot (LHF)—Women.

**Figure 9:**
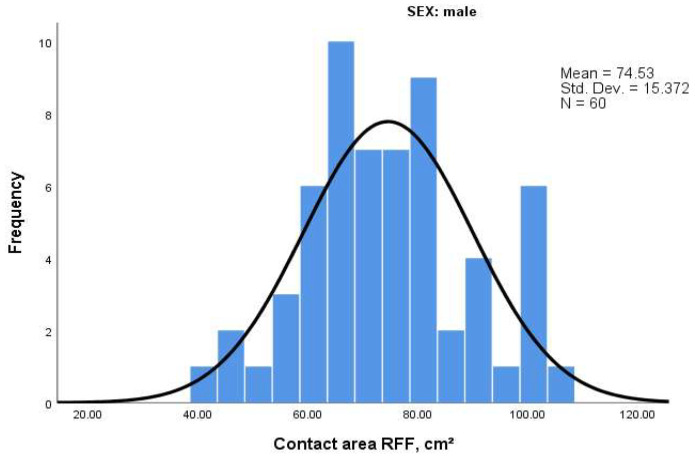
Histogram of Contact Area in Right Forefoot (RFF)—Men.

To further explore the distribution characteristics of baropodometric parameters, we analyzed skewness (symmetry of the distribution) and kurtosis (peakedness). Results are summarized in Table 10 and histograms are shown in Figure 8 and Figure 9.

For men, most parameters exhibited near-normal distributions, particularly for contact area and average load. Only the right forefoot (RFF) pressure showed moderate positive skewness (0.583), suggesting slightly higher values than low ones.

In contrast, women exhibited greater variability. Notably:

- Load in RFF showed high kurtosis (3.091), indicating a peaked distribution with values concentrated around the mean.
- Average pressure in LHF displayed positive skewness (1.71) and high kurtosis (6.418), suggesting more extreme values on the higher end and a sharper peak.

These differences may reflect greater biomechanical variability or compensatory patterns in the female subgroup, especially in the hindfoot.

### 3.9. Correlation Analysis

To explore the relationship between anthropometric characteristics and baropodometric parameters, Pearson correlation coefficients (r) were calculated separately for male and female participants. The goal was to identify which physiological variables most significantly influence plantar loading patterns.

In both sexes, body weight and BMI were strongly and positively correlated with multiple baropodometric indicators, most notably with load and contact area, particularly in the hindfoot. These findings are consistent with biomechanical expectations: individuals with greater mass exert more force and demonstrate wider plantar contact zones.

#### 3.9.1. Male Participants

Among men, weight was significantly correlated with hindfoot contact area and load:

- Left hindfoot contact area (r = 0.719, *p* < 0.001).
- Right hindfoot load (r = 0.713, *p* < 0.001).
- Left hindfoot load (r = 0.735, *p* < 0.001).

Similarly, BMI showed strong positive associations with the same hindfoot parameters (r > 0.66, *p* < 0.001). Although height had some moderate correlations with contact area in the forefoot (r ≈ 0.36), these were less consistent across all variables. Age was not significantly associated with any baropodometric measure.

Interestingly, average plantar pressure in the forefoot also correlated with body weight and BMI (*p* < 0.05), while maximum peak pressure did not, indicating that localized high-pressure points are less dependent on mass and more likely related to functional or structural biomechanical factors.

#### 3.9.2. Female Participants

In women, the correlation profile was similar but even stronger in magnitude:

- Hindfoot load had very strong correlations with both weight (r = 0.885) and BMI (r = 0.837).
- Moderate correlations were also observed for the forefoot region.

Average plantar pressure, particularly in the RFR, showed significant relationships with both weight (r = 0.555) and BMI (r = 0.544). These associations suggest that, in women, excess body mass is more diffusely distributed across plantar surfaces, leading to elevated baseline pressure levels [34].

As in the male group, maximum peak pressure did not correlate strongly with any anthropometric parameter, further supporting the idea that these high-pressure zones are more influenced by factors such as gait abnormalities, postural asymmetry, or localized structural differences.

In summary, while total foot loading and contact area scale with weight and BMI, peak pressure variability is more individualized, potentially reflecting unique postural patterns or foot mechanics. These results underline the importance of including anthropometric parameters in clinical baropodometric evaluations, while also recognizing that high peak pressures may warrant gait analysis and orthopedic investigation, especially when not directly explained by body mass. All correlation coefficients and significance levels are detailed in Table 11 and Table 12.

## 4. Discussion

The present study provides a comprehensive evaluation of plantar pressure distribution in a young, healthy population under static conditions using the Spine 3D Sensor Medica system. Through the analysis of contact area, load, average pressure, and maximum peak pressure, our results offer valuable normative data and confirm several biomechanical trends described in the literature—while also revealing previously underexplored asymmetries and sex-specific differences.

### 4.1. Summary and Interpretation of Findings

Our analysis confirms the physiological predominance of hindfoot loading during upright stance, with approximately 60% of body weight distributed over the rearfoot and 40% over the forefoot in both sexes. These findings are consistent with biomechanical models of postural alignment [35,36,37,38]. The higher average contact area and load in the hindfoot further supports this functional role.

Men exhibited significantly higher absolute values in most baropodometric parameters (contact area, load, average pressure), especially in the hindfoot, likely reflecting their greater body mass and stature. However, women displayed greater inter-limb asymmetries, particularly in the forefoot and hindfoot contact areas. These imbalances, while often subtle, may signal compensatory mechanisms linked to limb dominance, foot morphology, or mild postural deviations [39].

Although mean plantar pressures were higher in men at the forefoot level, maximum peak pressures did not differ significantly between sexes except in the right forefoot. This suggests that peak pressure hotspots are not solely determined by body mass, but rather by localized structural or functional characteristics such as foot arch type, heel-strike behavior, or muscular imbalances [40,41,42,43].

### 4.2. Correlation with Anthropometric Variables

A major strength of this study is the in-depth correlation analysis between anthropometric variables (age, weight, height, BMI) and baropodometric parameters. In both sexes, body weight and BMI were strongly and positively correlated with total load and contact area, especially in the hindfoot. These results are in line with those who observed increased plantar contact and load in overweight individuals [44].

Conversely, maximum peak pressure did not show significant correlations with weight or BMI. This reinforces the notion that focal overloads are more biomechanically than anthropometrically driven, and that static baropodometry may capture pressure irregularities not explainable solely by body size [45,46].

Women demonstrated slightly stronger correlations between BMI and pressure metrics than men, particularly in the right forefoot. This may reflect gender-specific differences in fat distribution, postural alignment, or foot structure, and supports the need for sex-stratified normative values in plantar pressure studies.

### 4.3. Comparison with Existing Literature

Our results differ from some previous studies in terms of absolute values, particularly for pressure measurements, which were lower than those previously reported [47,48,49]. These differences may be attributed to variations in platform characteristics (e.g., number of sensors, sampling frequency), testing protocols, or demographic profiles. The Spine 3D Sensor Medica system used in our study offers high resolution (4 sensors/cm^2^) and reliability, which may enhance sensitivity for detecting small inter-limb differences and subtler variations in pressure [50,51,52,53,54].

In contrast [55,56], who reported higher forefoot pressure in women possibly due to high-heel usage, our data suggest a more balanced distribution, perhaps owing to the younger and physically active cohort in our sample. This discrepancy reinforces the importance of context-specific reference values, especially in static analyses.

### 4.4. Clinical and Preventive Relevance

Understanding how body weight, BMI, and sex influence plantar pressure is crucial in clinical biomechanics, particularly for early detection of postural imbalances, custom orthotic design, and rehabilitation planning. The observed asymmetries, while mostly within acceptable physiological thresholds, warrant attention in overweight individuals, where excessive load concentration may predispose musculoskeletal conditions such as plantar fasciitis, Achilles tendinopathy, or knee pain [57,58,59,60,61].

Additionally, the identification of asymmetrical pressure patterns—not attributable to BMI—emphasizes the role of individualized assessments, including gait analysis, structural evaluation, and postural screening, particularly when prescribing interventions such as foot orthoses or corrective exercise [62,63,64,65].

Our results also support the growing recognition of baropodometry as a preventive tool in sports science and education, where postural screening in asymptomatic young adults may highlight compensatory mechanisms that, if unaddressed, could evolve into dysfunctions over time [66,67,68,69,70].

### 4.5. Limitations

Despite its strengths, this study has several limitations:

- Static-only analysis: While static baropodometry offers controlled and reproducible insights into plantar distribution, it cannot fully capture the complexities of dynamic loading during gait or functional movement. Future studies should incorporate dynamic assessments [71].
- Homogeneous sample: Participants were healthy, young adults with relatively narrow age and BMI ranges. Results may not generalize to elderly populations, children, or individuals with orthopedic or neurological impairments [72,73].
- Lack of foot morphology data: Parameters such as arch type, foot length, or pronation/supination were not measured but may significantly influence plantar loading.
- No dominant foot analysis: The study did not control or analyze foot dominance, which may influence asymmetry in loading patterns.

### 4.6. Future Directions

To build upon these findings, we propose:

1. Dynamic baropodometric evaluation during walking or sport-specific activities to identify functional abnormalities not observable under static conditions.
2. Expansion to clinical populations (e.g., obese, elderly, diabetic, orthopedic patients) to understand pathological pressure patterns and support therapeutic decision-making.
3. Validation across different platforms, to promote standardization and comparability of baropodometric findings in research and clinical settings [74].

## 5. Conclusions

This study offers a detailed analysis of static plantar pressure distribution in a young, healthy adult population using the Spine 3D Sensor Medica system. The findings confirm several fundamental biomechanical patterns—such as greater loading in the hindfoot compared to the forefoot—and reveal statistically significant differences between sexes, as well as notable inter-limb asymmetries.

Men exhibited higher absolute values for most baropodometric parameters, especially in the hindfoot, while women demonstrated greater variability and asymmetry, particularly in forefoot loading. These differences likely reflect variations in body structure, weight distribution, and possibly foot posture or habitual stance mechanics.

Body weight and BMI emerged as key determinants of plantar loading and contact area, particularly in the hindfoot, with strong positive correlations observed in both sexes. However, maximum peak pressures did not correlate significantly with anthropometric measures, suggesting a stronger influence from local biomechanical factors, such as gait anomalies or structural misalignments.

Importantly, individual-level analysis revealed significant asymmetries in several participants, especially those classified as overweight or obese. These imbalances may predispose individuals to conditions such as plantar fasciitis, chronic ankle instability, or metatarsalgia—highlighting the clinical value of early baropodometric screening.

The use of advanced baropodometric platforms, like the Spine 3D system, proves effective for identifying asymmetrical loading patterns and for supporting personalized interventions in rehabilitation, orthotic prescription, and postural correction. These findings underscore the importance of including plantar pressure assessments in routine musculoskeletal evaluations, particularly for individuals at risk of overuse injuries or postural dysfunctions.

Future research should focus on dynamic baropodometric evaluations and extend to broader populations to better understand the complex interactions between body structure, movement, and plantar mechanics.

## Figures and Tables

**Table 1:** Descriptive statistics of anthropometric parameters by sex (mean ± SD; range).

Parameter	Women (*n* = 53)	Men (*n* = 60)	Total (*n* = 113)
Age (years)	21.15 ± 3.97 (18–35)	21.00 ± 2.33 (18–34)	21.07 ± 3.19 (18–35)
Weight (kg)	67.83 ± 17.52 (45–108)	76.90 ± 12.06 (54–105)	72.65 ± 15.35 (45–108)
Height (m)	1.65 ± 0.06 (1.55–1.82)	1.79 ± 0.07 (1.65–1.97)	1.73 ± 0.10 (1.55–1.97)
BMI (kg/m^2^)	24.74 ± 6.05 (16.73–41.8)	23.99 ± 3.52 (17.17–34.68)	24.34 ± 4.87 (16.73–41.8)

**Table 2:** Description of baropodometric parameters recorded during static assessment.

Parameter	Unit	Description/Clinical Significance
Contact area	cm^2^	Surface area of the foot in contact with the platform; reflects the distribution of plantar surface load.
Absolute load	kgf ^1^	Total force applied by the foot in static position; used to evaluate weight-bearing asymmetries.
Relative load	% of body weight	Load distribution relative to total body mass; useful in assessing load imbalance.
Average pressure	kPa ^2^	Mean pressure applied on the surface of the foot; helps identify general stress zones.
Maximum peak pressure	kPa	Highest localized pressure point on the foot; relevant for identifying high-risk zones for injury.

^1^ kilogram-force; ^2^ kilopascals.

**Table 3:** Interpretation of asymmetry index (AI) values.

AI Value (%)	Interpretation	Clinical Relevance
<10%	Acceptable asymmetry	Considered normal and not clinically concerning.
10–20%	Moderate asymmetry	May suggest mild imbalance; monitor in follow-up.
>20%	Significant asymmetry	Indicates possible structural/functional imbalance

**Table 4:** Descriptive statistics of anthropometric parameters by sex.

Contact Area [cm^2^]	Women (*n* = 53)			Men (*n* = 60)			Total (*n* = 113)		
	Mean	SD	Range	Mean	SD	Range	Mean	SD	Range
Age [years]	21.15	3.97	18–35	21.00	2.33	18–34	21.07	3.19	18–35
Weight [kg]	67.83	17.52	45–108	76.90	12.06	54–105	72.65	15.35	45–108
Height [m]	1.65	0.06	1.55–1.82	1.79	0.07	1.65–1.97	1.73	0.10	1.55–1.97
BMI [kg/m^2^]	24.74	6.05	16.73–41.8	23.99	3.52	17.17–34.68	24.34	4.87	16.73–41.8

**Table 5:** Baropodometric parameters by anatomical region (*n* = 113).

Baropodometric Parameters (*n* = 113)	Forefoot		Hindfoot	
	Right	Left	Right	Left
Contact area (cm^2^)				
Average value	73.64	70.00	88.19	81.26
SD	17.04	16.08	21.62	21.01
Variation range	41–126	33–125	37–145	38–146
Load (kgf/%)				
Average value	16.55/22.81	14.64/20.27	21.58/29.52	19.94/27.37
SD	4.52/4.43	3.82/3.90	6.19/4.79	5.67/4.48
Variation range	(7–33)/(11–38)	(7–26)/(12–30)	(9–43)/(19–40)	(10–37)/(14–38)
Average pressure (kPa)				
Average value	22.19	20.66	24.10	24.53
SD	4.09	3.71	4.35	5.43
Variation range	14–33	13–35	16–42	14–50
Maximum peak pressure (kPa)				
Average value	44.94	43.21	48.02	47.70
SD	8.26	7.59	9.14	9.56
Variation range	23–71	23–61	23–78	23–82

**Table 6:** Comparison of contact area by foot region and sex.

Contact Area [cm^2^]	Women (*n* = 53)			Men (*n* = 60)			*p*-Value
	Mean	SD	Range	Mean	SD	Range	
Right forefoot (RFF)	72.62	18.84	42–126	74.53	15.37	41–105	0.554
Left forefoot (LFF)	67.36	17.46	33–125	72.33	14.50	37–101	0.101
Right hindfoot (RHF)	82.34	22.07	37–145	93.35	19.99	46–131	0.006
Left hindfoot (LHF)	74.53	21.21	38–146	87.20	19.10	45–125	0.001

**Table 7:** Absolute load (kgf) distribution by sex and anatomical region.

Load [kgf]	Women (*n* = 53)			Men (*n* = 60)			*p*-Value
	Mean	SD	Range	Mean	SD	Range	
RFF	15.66	4.76	7–33	17.33	4.19	7–31	0.006
LFF	13.53	3.57	7–22	15.62	3.79	7–26	0.004
RHF	20.21	6.77	9–35	22.78	5.41	12–37	0.007
LHF	18.57	6.29	10–26	21.15	4.80	11–32	0.002

**Table 8:** Average pressure distribution across foot regions and sexes.

Load [kgf]	Women (*n* = 53)			Men (*n* = 60)			*p*-Value
	Mean	SD	Range	Mean	SD	Range	
RFF	21.28	4.22	14–33	23.00	3.83	16–32	0.030
LFF	19.92	3.10	14–26	21.32	4.09	13–35	0.046
RHF	24.08	5.00	16–42	24.12	3.74	18–36	0.575
LHF	24.83	5.73	14–50	24.27	5.19	14–41	0.487

**Table 9:** Maximum peak pressure distribution.

Load [kgf]	Women (*n* = 53)			Men (*n* = 60)			*p*-Value
	Mean	SD	Range	Mean	SD	Range	
RFF	42.98	8.40	23–67	46.67	7.80	31–71	0.017
LFF	41.96	7.24	23–59	44.32	7.78	27–61	0.100
RHF	46.36	9.63	23–78	49.48	8.49	34–67	0.051
LHF	46.85	10.66	23–82	48.45	8.50	34–68	0.246

**Table 10:** Skewness and Kurtosis for Baropodometric Variables.

Baropodometric Parameter	Men		Women	
	Skewness	Kurtosis	Skewness	Kurtosis
Contact Area				
RFF	42.98	8.40	23–67	0.017
LFF	41.96	7.24	23–59	0.100
RHF	46.36	9.63	23–78	0.051
LHF	46.85	10.66	23–82	0.246
Load				
RFF	0.371	1.680	1.452	3.091
LFF	−0.019	0.585	0.474	−0.476
RHF	0.308	−0.561	1.015	1.348
LHF	0.088	−0.697	1.050	0.879
Average plantar pressure				
RFF	0.614	−0.235	0.386	−0.254
LFF	0.558	0.914	−0.124	−0.896
RHF	0.650	0.854	1.143	2.209
LHF	1.036	2.055	1.710	6.418
Maximum peak plantar pressure				
RFF	0.583	0.390	0.191	0.457
LFF	0.225	−0.308	−0.010	0.063
RHF	0.354	−0.720	0.842	1.849
LHF	0.413	−0.672	0.865	1.942

**Table 11:** Correlations between baropodometric and anthropometric parameters—men.

Parameter		Contact Area				Load			
		RFF	LFF	RHF	LHF	RFF	LFF	RHF	LHF
Age	Correlation coefficient	−0.096	0.003	−0.032	0.042	−0.078	0.075	−0.139	0.008
	Sig. (2-tailed)	0.497	0.985	0.810	0.748	0.553	0.570	0.291	0.954
Weight	Correlation coefficient	0.416 **	0.408 **	0.689 **	0.719 **	0.576 **	0.604 **	0.713 **	0.735 **
	Sig. (2-tailed)	0.001	0.001	0.000	0.000	0.000	0.000	0.000	0.000
Height	Correlation coefficient	0.363 **	0.367 **	0.254	0.339 **	0.375 **	0.360 **	0.164	0.236
	Sig. (2-tailed)	0.004	0.004	0.050	0.008	0.003	0.005	0.209	0.070
BMI	Correlation coefficient	0.262 *	0.255 *	0.617 **	0.594 **	0.417 **	0.457 **	0.684 **	0.660 **
	Sig. (2-tailed)	0.043	0.049	0.000	0.000	0.001	0.000	0.000	0.000
**Parameter**		**Average Plantar Pressure**				**Maximum Peak Pressure**			
		**RFF**	**LFF**	**RHF**	**LHF**	**RFF**	**LFF**	**RHF**	**LHF**
Age	Correlation coefficient	0.283 *	0.308 *	0.030	−0.057	0.246	0.192	0.041	−0.005
	Sig. (2-tailed)	0.029	0.017	0.822	0.667	0.058	0.141	0.753	0.971
Weight	Correlation coefficient	0.283 *	0.308 *	0.030	−0.057	0.246	0.192	0.041	−0.005
	Sig. (2-tailed)	0.029	0.017	0.822	0.667	0.058	0.141	0.753	0.971
Height	Correlation coefficient	0.075	0.112	−0.112	−0.131	0.147	0.024	0.040	−0.044
	Sig. (2-tailed)	0.568	0.394	0.395	0.318	0.263	0.856	0.759	0.740
BMI	Correlation coefficient	0.254	0.261 *	0.083	0.001	0.174	0.183	0.007	0.000
	Sig. (2-tailed)	0.051	0.044	0.529	0.993	0.183	0.161	0.957	0.999

* Statistically significant value, *p* < 0.05. ** Extremely statistically significant value, *p* < 0.01.

**Table 12:** Correlations between baropodometric and anthropometric parameters—women.

Parameter		Contact Area				Load			
		RFF	LFF	RHF	LHF	RFF	LFF	RHF	LHF
Age	Correlation coefficient	0.065	0.134	0.105	0.127	0.019	0.108	−0.044	−0.050
	Sig. (2-tailed)	0.643	0.341	0.456	0.367	0.892	0.440	0.754	0.724
Weight	Correlation coefficient	0.393 **	0.401 **	0.741 **	0.737 **	0.734 **	0.685 **	0.885 **	0.842 **
	Sig. (2-tailed)	0.004	0.003	0.000	0.000	0.000	0.000	0.000	0.000
Height	Correlation coefficient	0.028	0.083	0.316 *	0.284 *	0.101	0.280 *	0.272 *	0.318 *
	Sig. (2-tailed)	0.843	0.553	0.021	0.039	0.473	0.042	0.049	0.020
BMI	Correlation coefficient	0.407 **	0.395 **	0.666 **	0.676 **	0.747 **	0.636 **	0.837 **	0.780 **
	Sig. (2-tailed)	0.003	0.003	0.000	0.000	0.000	0.000	0.000	0.000
**Parameter**		**Average Plantar Pressure**				**Maximum Peak Pressure**			
		**RFF**	**LFF**	**RHF**	**LHF**	**RFF**	**LFF**	**RHF**	**LHF**
Age	Correlation coefficient	−0.023	−0.030	−0.184	−0.204	0.015	0.018	−0.134	−0.128
	Sig. (2-tailed)	0.869	0.829	0.187	0.144	0.915	0.897	0.340	0.361
Weight	Correlation coefficient	0.555 **	0.425 **	0.441 **	0.209	0.458 **	0.316 *	0.214	0.090
	Sig. (2-tailed)	0.000	0.002	0.001	0.132	0.001	0.021	0.124	0.523
Height	Correlation coefficient	0.145	0.278 *	0.093	0.112	0.182	0.295 *	0.152	0.160
	Sig. (2-tailed)	0.301	0.044	0.507	0.426	0.191	0.032	0.277	0.252
BMI	Correlation coefficient	0.544 **	0.367 **	0.440 **	0.189	0.439 **	0.254	0.182	0.048
	Sig. (2-tailed)	0.000	0.007	0.001	0.175	0.001	0.066	0.193	0.735

* Statistically significant value, *p* < 0.05. ** Extremely statistically significant value, *p* < 0.01.

## Data Availability

The data presented in this study is available on request from the corresponding author. The data is not publicly available due to privacy and ethical restrictions.

## Abbreviations

The following abbreviations are used in this manuscript:

AI	Asymmetry Index
ANOVA	Analysis of Variance
BMI	Body Mass Index
cm^2^	Square centimeters
kPa	Kilopascal
kgf	Kilogram-force
LFF	Left Forefoot
LHF	Left Hindfoot
RFF	Right Forefoot
RHF	Right Hindfoot
SD	Standard Deviation
SPSS	Statistical Package for the Social Sciences
WHO	World Health Organization
